# Angiogenesis in Disease

**DOI:** 10.3390/ijms231810962

**Published:** 2022-09-19

**Authors:** Diego La Mendola, Maria Letizia Trincavelli, Claudia Martini

**Affiliations:** Department of Pharmacy, University of Pisa, Via Bonanno Pisano 6, 56126 Pisa, Italy

Angiogenesis is a multi-step process by which new blood capillaries are formed starting from preexisting functional vessels [1]. Angiogenesis is strictly regulated by the balance of many positive and negative angiogenic modulators within the vascular microenvironment [2].

There is accumulating evidence that many diseases are angiogenesis-dependent. Pathological angiogenesis is a hallmark of many cancers, diabetic retinopathy, autoimmune diseases, rheumatoid arthritis, atherosclerosis, cerebral ischemia, cardiovascular diseases and delayed wound healing [3,4].

Many efforts in this area of research are leading to the discovery of new biochemical pathways, pharmacological targets and of a growing number of pro- and anti-angiogenic molecules as potential drugs in such pathologies.

The aim of this Special Issue was to give an insight into the role of angiogenesis in different diseases, focusing on the signaling pathways of angiogenesis activators and inhibitors in physiological as well as pathological conditions.

The so-called “angiogenic switch” refers to a time-restricted event determined by a local change in the tissue environment, where the balance between pro-angiogenic and anti-angiogenic molecules tilts towards activators, resulting in the promotion of tissue vascularization. Balberova et al. review the latest studies on the angiogenic switch issue in the cerebrovascular and cardiovascular systems of athletes playing different sports, in particular aerobic ones such as long-distance running [5]. The imbalance between pro- and anti-angiogenic factors leads to a decrease in the functional resources of the brain, hearth and circulatory system, musculoskeletal tissues and respiratory system. A relevant point is the possible effects of angiogenic factors on the lung tissues, as there are many controversial questions about the role of growth factors in the physiology as well as pathology of the lungs. The action of vascular endothelial growth factor A (VEGF-A) in the physiological development of lung tissue is widely studied, but the influence of other growth factors such as platelet-derived growth factor (PDGF) remains to be fully elucidated [6]. An understanding of the training process is critical for maintaining the athletes’ physical health and to obtain information on potential related cardiovascular diseases.

PDGF is an angiogenic factor that regulates different biochemical steps. It is the major mitogen for connective tissue cells, stimulating cell survival, growth and proliferation, as well as changes in cell shape and motility [7]. Paolini et al. focus on the role of PDGF as a key signaling molecule in the onset of systemic sclerosis, a heterogeneous disorder of the connective tissue characterized by immune/inflammatory manifestations, vascular dysfunctions and organ fibrosis [8]. The authors highlight the role of PDGF in the activation of fibroblasts and in its upregulation in fibrotic dermal lesions of patients with scleroderma, paving the way toward novel therapeutic strategies directed toward this molecular target [9].

The angiogenic switch is often invoked in the development of many cancers. Indeed, the interaction between neoplastic cells and newly formed vessels is one of the fundamental biochemical pathways for the growth of solid tumors and metastases formation [10]. Vasquez et al. report on the neovascularization of glioblastoma, a primary brain cancer and one of the more aggressive tumors [11]. There is the involvement of widely studied angiogenic factors molecules such as VEGF and PDGF in cancer progression, but the authors discuss recent data which point to the protein netrin-1 as a non-canonical angiogenic biomolecule in the promotion of neovascularization processes in glioblastoma. In particular, netrin-1 expression has been detected in different human glioblastoma cell lines, including the highly aggressive and invasive U373MG and U251MG cell lines, and so represents a potential biomarker. The decrease in netrin-1 expression may also represent a novel approach in the treatment of glioblastoma improving patient survival.

The need to find specific biomarkers for an accurate diagnosis is highlighted in the work of Olivera et al. [12]. In this research article, the authors try to find molecular biomarker for a differential and more accurate diagnosis between adrenocortical adenomas and adrenocortical carcinomas. Furthermore, the same biomarker may differentiate adrenocortical carcinomas which present heterogeneity and different prognosis. The authors analyze the expression of different angiogenic factors as VEGF and angiopoietin (Ang) by means of immunohistochemistry on adrenal tissues from patients affected by adrenocortical carcinoma, adrenocortical adenoma with Cushing syndrome and non-functioning adrenocortical adenoma. A relevant result is the discovery that Ang-Tie pathway signaling molecules are expressed in adenocarcinoma cancers and are associated with greater vascular permeability contributing to tumor cell dissemination. Although none of these biomarkers are shown to be suitable for adenocarcinoma cancer diagnosis, the Ang–Tie pathway may represent a target in the development of new and innovative pharmaceutical approaches.

The identification of specific angiogenic pathways through which the tumor is vascularized allows us to design and synthesize new molecules able to inhibit these steps. In the communication by Schere-Levy et al., the activity of an heptapeptide encompassing the first seven residues of angiotensin II is tested on a mouse model of oral squamous cell carcinoma, the most common malignant cancer affecting the oral cavity [13]. The peptide angiotensin 1–17 is derived by the enzymatic activity of angiotensin-converting enzyme 2 (ACE 2), and mice treated with such biomolecule show a decrease in premalignant lesions in the oral mucosa accompanied by a significant reduction in proliferating cells. The peptide affects the PI3K/Akt/mTOR signaling route in oral papilloma, decreasing pS6 accumulation, an early event observed also in other squamous cell carcinoma [14]. Taking into account that the heptapeptide has already been approved by the Food and Drug Administration for use in various clinical trials [15], the authors suggest that its application in cancer treatment should be tested soon.

It is of note that ACE 2 allows severe acute respiratory syndrome coronavirus 2 (SARS-CoV-2) to enter the host cells, and consequently the virus multiplication and COVID-19’s progression. Dettlaff-Pokora and Swierczynski review how SARS-CoV-2 impairs the renin–angiotensin–aledosterone system via binding the ACE2 enzyme [16]. ACE2 plays a key role in the balance between angiotensin 2 and angiotensin (1–7), regulating blood pressure. The authors underline how disorders in the angiotensin metabolism may affect SARS-CoV-2 progression and how some drugs used as ACE2 inhibitors could increase infection risk, although further clinical observations and experimental studies are required.

The inhibition of angiogenic factors can be used in diseases where the excess of vascularization is a cause; on the other hand, the promotion of angiogenesis can be used in those pathologies where the promotion of new vessels can improve the survival of poorly perfused tissues [17].

In diabetes and vascular pathologies, chronic wound healing has become an increasingly relevant issue. Cucci et al. report on the role of angiogenin protein [18], a potent angiogenic factor that regulates the expression of different growth factors as VEGF [19], and its potential connection with copper ions. The metal is also an angiogenic factor that regulates different steps of angiogenesis including the wound healing process, and a correct restoration of copper homeostasis may represent a valuable pharmacological approach [20]. The authors report that copper regulates angiogenin expression and that the protein is able to bind copper ions [21,22]. Such binding affects the activity of protein, and the determination of copper/angiogenin biochemical pathways represents a potential therapeutic target in diseases characterized by a defect as well as excess in angiogenic processes.

The angiogenesis is also critical for successful fracture healing. Indeed, osteoporosis is a common musculoskeletal disease that affects the elderly population, and aging may have negative effects on angiogenesis at a fracture site, delaying fracture healing.

In their research article, Dadwal et al. studied the influence of endothelial cells and their angiogenic potential on bone healing [23]. Endothelial cells express sirtuin 1, a NAD^+^-dependent histone deacetylase, implicated in several biologic processes and able to counteract vascular aging [24]. Furthermore, SIRT1 via SRT1720 has been shown to increase bone mass and decrease age-dependent bone loss. The authors investigated the effect of SRT1720 treatment on endothelial cells function in aging, using bone marrow and lung endothelial cells derived from young and old mice.

The experimental data showed that treatment with SRT1720 significantly increased the proliferation of lung endothelial cells for all mice, whereas it increased the proliferation of bone marrow only for old females. It appears that the overall effect of SRT1720 is beneficial with regard to angiogenic potential, supporting SIRT activators as potential therapeutic options to improve angiogenesis and tissue regeneration in the context of aging.

Certainly, peripheral nerve repair and regeneration remain among the greatest challenges in tissue engineering and regenerative medicine [25]. There is a close interaction between the vascular and neural systems; necortical angiogenesis proceeds in a spatially and temporally restricted manner so as to build a specific vascular niche that supports ongoing neurogenesis during neocortical development [26]. Moreover, the neurotrophins display angiogenic activity, and angiogenic factors can act as neuron growth factors [27,28,29]. Peripheral nerves provide the path for all types of axons that comprise the peripheral nervous system, and the review by Saio et al. focuses on the roles of angiogenesis in the regeneration of peripheral nerves and on the latest therapeutic strategies for the treatment of peripheral nerve injury [30]. Among angiogenic factors, VEGF-A has more often been used in the treatment of peripheral nerve injury, but it is essential to also identify also extracellular components involved in vascularization in nerve regeneration to achieve the effective artificial control of angiogenesis.

Altogether, these new studies highlight once again the relevance of understanding angiogenesis pathways for the development of new therapies and drugs for a wide spectrum of diseases.
